# *Trypanosoma cruzi* infection, discrete typing units and feeding sources among *Psammolestes arthuri* (Reduviidae: Triatominae) collected in eastern Colombia

**DOI:** 10.1186/s13071-019-3422-y

**Published:** 2019-04-08

**Authors:** Natalia Velásquez-Ortiz, Carolina Hernández, Giovanny Herrera, Lissa Cruz-Saavedra, Adriana Higuera, Luisa M. Arias-Giraldo, Plutarco Urbano, Andrés Cuervo, Aníbal Teherán, Juan David Ramírez

**Affiliations:** 1 Grupo de Investigaciones Microbiológicas-UR (GIMUR), Programa de Biología, Facultad de Ciencias Naturales y Matemáticas, Universidad del Rosario, Bogotá, Colombia; 2 Grupo de Investigaciones Biológicas de la Orinoquia, Fundación Universidad del Trópico Americano (Unitrópico), Yopal, Colombia; 3 Secretaría Departamental de Salud de Arauca, Arauca, Colombia; 4 Grupo de Investigación COMPLEXUS, Fundación Universitaria Juan N. Corpas, Bogotá, Colombia

**Keywords:** Chagas disease, *Trypanosoma cruzi*, *Psammolestes arthuri*, Colombia

## Abstract

**Background:**

Chagas disease (CD) is caused by the protozoan parasite *Trypanosoma cruzi*, and is transmitted by hematophagous insects of the family Reduviidae. *Psammolestes arthuri* is a sylvatic triatomine distributed in Colombia and Venezuela which feeds on birds and there are a few studies that have reported *Ps. arthuri* naturally infected with *T. cruzi*. In Colombia, *Ps. arthuri* has been found in dwellings, making it important to evaluate its possible role in the *T. cruzi* transmission cycle. We aimed to evaluate the presence of *T. cruzi* and feeding sources of *Ps. arthuri* to elucidate new possible scenarios of *T. cruzi* transmission in the country.

**Methods:**

A total of 60 *Ps. arthuri* were collected in Arauca and Casanare, Colombia. We detected and genotyped *T. cruzi* and identified feeding sources. The frequency of the presence of *T. cruzi* was obtained and compared with different eco-epidemiological variables. Multiple correspondence analysis was conducted to explore associations between eco-epidemiological variables and the presence of *T. cruzi*; with these results, a logistic regression was used to determine statistical associations.

**Results:**

The infection rate of *T. cruzi* was 70.7% and was mostly associated with insect stage, sex, bird nest and feeding source. Regarding discrete typing units (DTUs), TcI was found in 54.7% samples, of which 21.7% (5/23) were TcI_Dom_, 52.1% (12/23) had mixed infection (TcI_Dom_-TcI_Sylv_), and single infection with TcI_Sylv_ was not detected. Mixed infections (TcI/TcII-TcVI) were found in 9.52% (4/42) of the samples; of these, 14.2% (6/42) were TcII-TcVI. A total of 15 feeding sources were identified and the most frequent were: *Cranioleuca baroni* (35.85%), *Homo sapiens* (26.42%), *Thraupis episcopus* (11.32%) and *Serinus albogularis* (3.77%).

**Conclusions:**

Although *Ps. arthuri* is mainly ornithophilic, this species may be feeding on other animals that can be infected with *T. cruzi*, possibly playing a role maintaining the zoonotic cycle of the parasite. Further studies with molecular techniques and wider sampling are needed to improve information regarding infection rates, ecotopes and habits with the aim of evaluating whether *Ps. arthuri* could be a potential *T. cruzi* vector.

**Electronic supplementary material:**

The online version of this article (10.1186/s13071-019-3422-y) contains supplementary material, which is available to authorized users.

## Background

*Trypanosoma cruzi* is a flagellated protozoan that causes Chagas disease (CD), and is mainly transmitted by insects of the family Reduviidae (Order: Hemiptera) through their feces [1]. CD affects about 6–7 million people around the world and is especially important in Latin America, where it is considered a public health problem [2]. Based on 2010 estimates, in Colombia there are 437,960 infected people, with an estimated 5274 new cases annually due to vectorial transmission [3]. CD has been reported in different geographical regions of the country, with the departments of Casanare, Arauca, Santander, Boyacá, Norte de Santander and Cundinamarca being endemic for the disease [4]. Due to the high genetic diversity of *T. cruzi*, the parasite has been subdivided into discrete typing units (DTUs): TcI to TcVI [5, 6] and a genotype associated to bats (TcBat) [7–9]. Each DTU presents different characteristics based on its geographical distribution, clinical manifestations of the disease and epidemiological associations. TcI is the most widely distributed DTU in the Americas, and because of its genetic diversity, it has been divided into domestic and sylvatic genotypes (TcI_Dom_ and TcI_Syl_) [10].

Triatomines of the family Reduviidae are widely distributed in the Americas, mainly in the Neotropical region at different altitudes, they present a high adaptation to different feeding sources, especially sylvatic and domestic mammals [11, 12]. There are about 180 species of synantropic, sylvatic and domestic animals that have been reported as potential *T. cruzi* reservoirs, mainly rodents and marsupials (Didelphimorphia) [13], rodents, bats, dogs, birds (hens), cows, armadillos and other mammals (anteaters and humans) [13–16]. In Colombia there are 26 registered triatomine species of which 15 have been reported as naturally infected with *T. cruzi* [17, 18]. *Rhodnius prolixus* and *Triatoma dimidiata* are the main species that transmit *T. cruzi* and are considered primary vectors of the parasite; therefore, surveillance programs focus mainly on these two species [15, 18]. Recently, some species, such as *Panstrongylus geniculatus*, *R. pallescens* and *Eratyrus mucronatus*, have begun to be considered important in the transmission of this parasite because of their domiciliation process due to deforestation and urbanization [18, 19] that disturbs triatomine ecology [11, 15]. Furthermore, defaunation caused by ecological disturbances, such as anthropogenic deforestation and land-use conversion, directly impacts biotic interactions [20]. In this case, fauna migration and extinction can indirectly affect triatomine populations due to their hematophagic habits, forcing them to find other ecosystemic units and feeding sources.

There are some triatomine species considered as secondary vectors for the transmission of *T. cruzi* to humans, which include *Psammolestes arthuri*, *Panstrongylus lignarius*, *Cavernicola pilosa*, *Belminus rugulosus*, *Rhodnius colombiensis*, *Triatoma dispar* and *Microtriatoma trinidadensis*. *Psammolestes arthuri* is a sylvatic triatomine distributed in Colombia and Venezuela which feeds mainly on birds, and is frequently found in nests [21]. There are a few studies that report *Ps. arthuri* naturally infected with *T. cruzi* [22–24]. One study conducted in Venezuela in 2014 by Cruz-Guzmán et al. [25] reported *Ps. arthuri* naturally infected with *T. cruzi* and coexisting in the same geographical space with *R. prolixus* and *T. maculata*, but it is still not considered important in the transmission of *T. cruzi* for humans [26]. Despite of this, due to recent evidence in Venezuela, the possibility that *Ps. arthuri* is feeding on infected mammals thus contributing to the maintenance of the sylvatic cycle of *T. cruzi* [25, 27] in Colombia cannot be dismissed. In Colombia, *R. prolixus*, *E. mucronatus* and *Ps. arthuri* have been reported inside houses, associated with the nearby presence of *Attalea butyracea* [28, 29]. This fact is relevant because *Ps. arthuri* could be participating in the domestic cycle of *T. cruzi*. Then, considering the ability of triatomines to be adaptive depending on the feeding sources available, it is important to include sylvatic triatomines in eco-epidemiology studies of CD [30] because they can represent a connection between domestic and sylvatic transmission cycles of the parasite [15].

Therefore, in the light of the current absence of studies investigating the plausible presence of *T. cruzi* in sylvatic triatomines such as *Ps. arthuri*, we evaluated the presence of *T. cruzi*, the DTUs and feeding sources of *Ps. arthuri* collected in the departments of Arauca and Casanare, Colombia (eastern departments of the country), with the aim to elucidate new possible scenarios of *T. cruzi* transmission in the country.

## Methods

### Sampling, DNA extraction and detection of *T. cruzi*

**Fig. 1:**
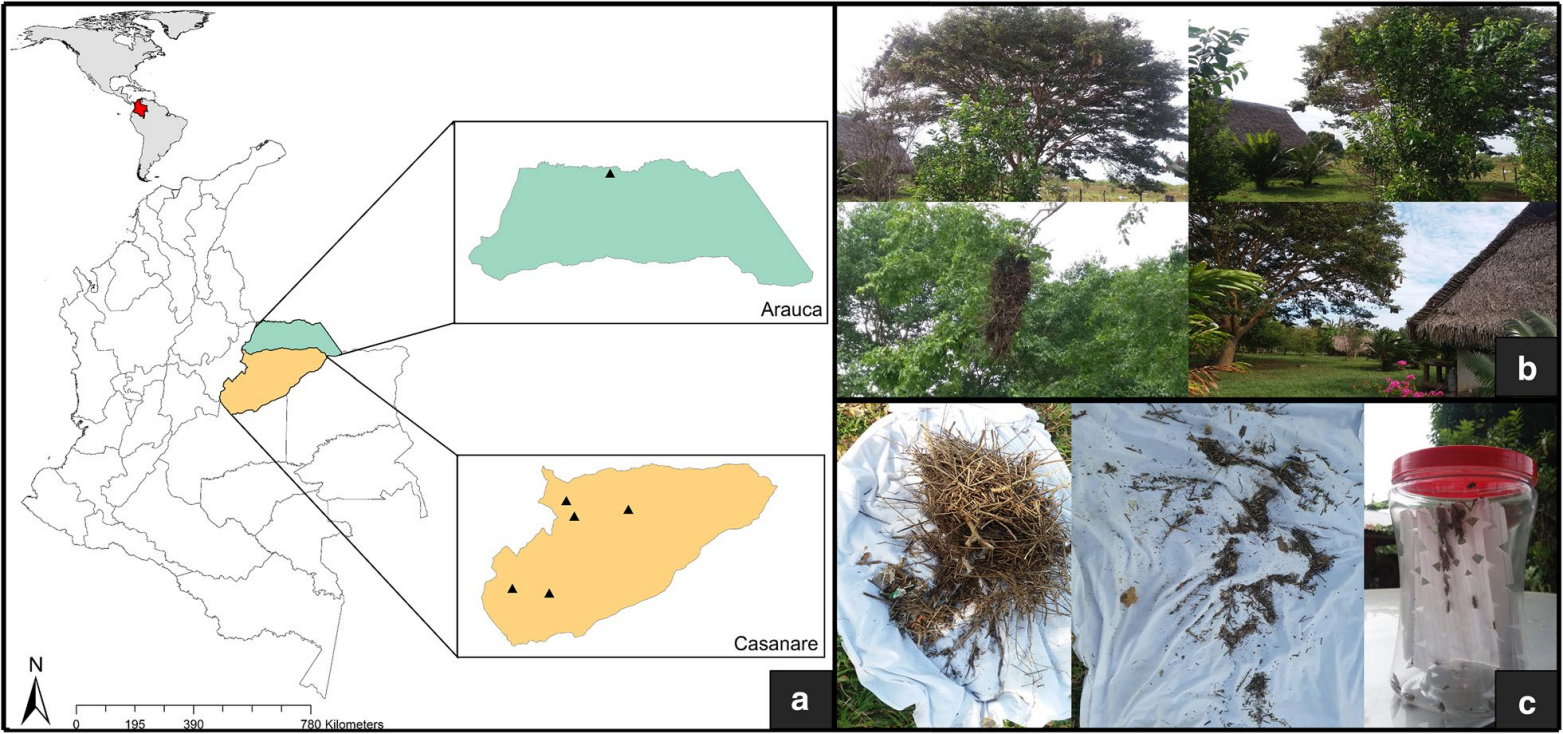
**a** Geographical distribution of 60 *Ps. arthuri* collected in the departments of Arauca and Casanare, Colombia. **b** Collection sites in Casanare. **c** Capturing methods in the field

A total of 60 *Ps. arthuri* were collected in the departments of Arauca (2 specimens) and Casanare (58 specimens) (Fig. 1a). In Casanare, specimens were collected in the municipalities of Mani (Mararabe: 4°49′59.88″N, 72°20′60″W), Monterrey (Marenao: 4°53′4.0992″N, 72°45′19.08″W), Paz de Ariporo (Caño chiquito: 5°45′0″N, 71°28′59.988″W), Pore (El verde: 5°40′34.1976″N, 72°4′31.0368″W) and Tamara (La picacha: 5°50′40.632″N, 72°9′49.176″W). In Arauca, the specimens were collected in Arauquita (Arauca: 6°58′5.8512″N, 71°11′47.6124″W). Casanare specimens were collected in trees near human houses (Fig. 1b) and captured in *Cacicus cela* (yellow-rumped cacique) and *Phacellodomus rufifrons* (rufous-fronted thornbird) nests using manual capturing (Fig. 1c). Furthermore, Arauca insects were collected in the domestic ecotope (inside the houses). All specimens were stored and conserved in ethanol until processing. DNA extraction of the insects’ guts was conducted using a Qiagen Dneasy Blood & Tissue kit (Qiagen, Berlin, Germany). Detection of *T. cruzi* was conducted by end-point qPCR using TaqMan Fast Advanced Master Mix 2× (Roche Diagnostics GmbH, Mannheim, Germany), water and the primers cruzi1 (10 µM) (5′-AST CGG CTG ATC GTT TTC-3′), cruzi2 (10 µM) (5′-AAT TCC TCC AAG CAG CGG ATA-3′) and a cruzi3 probe (5 µM) (FAM-CAC ACA CTG GAC ACC AA-NFQ-MGB) to detect the satellite tandem repeat DNA of the parasite (166 bp) following the conditions previously reported [31]. A Ct value < 38 was considered as positive amplification [15]. For insects with a positive qPCR result, a conventional PCR for kinetoplast DNA amplification was conducted using Buffer Taq 10×, MgCl_2_ 50 mM, dNTPs 25 mM, Taq Platinum 5 U/µl, water and the primers 121 (50 pmol/µl) (5′-AAA TAA TGT ACG GGK GAG ATG CAT GA-3′) and 122 (50 pmol/µl) (5′-GGT TCG ATT GGG GTT GGT GTA ATA TA-3′) to discriminate between *T. cruzi* (330 bp) and *T. rangeli* (400–450 bp) as reported elsewhere [32].

### Genotyping of *T. cruzi*

Parasite genotyping was accomplished by the amplification of the spliced leader intergenic region of miniexon gene (SL-IR), dividing DTUs in two groups: TcI (350 bp) and TcII-TcVI (300 bp). The reaction mix consisted of Go Taq Green Master Mix 2×, water and primers TCC (10 nM) (5′-CCC CCC TCC CAG GCC ACA CTG-3′), TC1 (10 nM) (5′-GTG TCC GCC ACC TCC TTC GGG CC-3′) and TC2 (10 nM) (5′-CCT GCA GGC ACA CGT GTG TGT G-3′) [33]. Then, insects with mixed infection (TcI/TcII-TcVI) and TcII-TcVI group results were submitted to Sanger sequencing using the TCC primer as reported elsewhere [34]. PCR products were cleaned using ExoSAP-IT^®^ Express PCR Product Cleanup 75001/75002 (Affymetrix, USB, California, USA) and then submitted to Sanger sequencing. Sequences were aligned in MEGA X software [35] and submitted to BLASTn for similarity search.

### Feeding source characterization

A 215 bp fragment of the *12S* gene fragment was amplified using Go Taq Green Master Mix, water and primers L1085 (10 nM) (5′-CCC AAA CTG GGA TTA GAT ACC C-3′) and H1259 (10 nM) (5′-GTT TGC TGA AGA TGG CGG TA-3′) as reported by Dumonteil et al. [35]. PCR products were cleaned using ExoSAP-IT^®^ Express PCR Product Cleanup 75001/75002 (Affimetrix) and then submitted to Sanger sequencing. Resulting sequences were edited in MEGA X software [36] and submitted to BLASTn for similarity search.

### Statistical analysis

We determined the frequency of *T. cruzi* infection, as well as DTUs and feeding sources, regarding variables such as ecotope (sylvatic or domestic), insect stage (adult or nymph), sex (male or female) and bird nests (*P. rufifrons* or *C. cela*) to find any plausible associations among them. We carried out a multiple correspondence analysis (MCA) using 3 dimensions to explore the proximity of the variables with the presence of *T. cruzi*. The MCA was made with 51 samples, since 9 samples were excluded due to their lack of information for the evaluated variables. Cronbach’s alpha coefficient and inercy were calculated to establish dimension consistency in the model and to determine the proportion of total variability contributed by each variable in the matrix, respectively.

A binary logistic regression, without including the intercept, was made to determine the statistical associations between *T. cruzi* infection and the explicative variables: locality, ecotope, insect stage, sex, bird nest and feeding source (*x*_*1*_, *x*_*2*_, *x*_*3*_….), previously identified in the MCA. Logistic regression was selected between five multivariate models that adjusted to type of data (Additional file 1: Figure S1). Statistical analyses were performed in Statistical Package for the Social Sciences (SPSS) v.25 and a *P*-value < 0.05 was considered statistically significant.

## Results

### Frequency of *T. cruzi* infection and DTUs

Of the 60 *Ps. arthuri* collected, we found that 70.7% (42/60) were positive by end-point qPCR for *T. cruzi*. Of these, TcI was found in 54.7% of the samples (23/42), of which 21.7% (5/23) were TcI_Dom_, 52.1% (12/23) had mixed infection (TcI_Dom_-TcI_Sylv_) and 26% (6/23) were not able to be typed. Mixed infection (TcI/TcII-TcVI) was found in 9.52% (4/42) of the samples, 14.2% (6/42) were TcII-TcVI and 21.4% (9/42) samples were not able to be typed. Table 1 shows the eco-epidemiological information evaluated by ecotope, insect stage, sex and bird nests. As shown in Table 1, 97.6% of the *Ps. arthuri* were positive for *T. cruzi* belonging to sylvatic ecotope, whereas just one domestic insect was positive for *T. cruzi* presence. No nymphs were infected with *T. cruzi* and 70% of the insects were found in *Phacellodomus rufifrons* nests. No insects were found infected with *T. rangeli.*

**Table 1:** Frequency of infection with *T. cruzi* among eco-epidemiological variables

Eco-epidemiological variable	*T. cruzi*		
	Positive (%)	Negative (%)	All (95% CI)
Ecotope			
Sylvatic	41 (97.6)	17 (94.4)	58 (89.7–99.3)
Domestic	1 (2.4)	1 (5.6)	2 (0.7–10.3)
Insect stages			
Adults	42 (100)	15 (83.3)	57 (87.3–98.6)
Nymphs	0 (0)	3 (16.7)	3 (1.4–12.7)
Sex			
Male	22 (52.4)	7 (46.7)	29 (38.1–63.5)
Female	20 (47.6)	8 (53.3)	28 (16.0–38.1)
Bird nest			
*Phacellodomus rufifrons*	29 (70.7)	14 (82.4)	43 (61.9–84.0)
*Cacicus cela*	12 (29.3)	3 (17.6)	15 (16.0–38.1)

### Feeding sources of *Ps. arthuri*

**Fig. 2:**
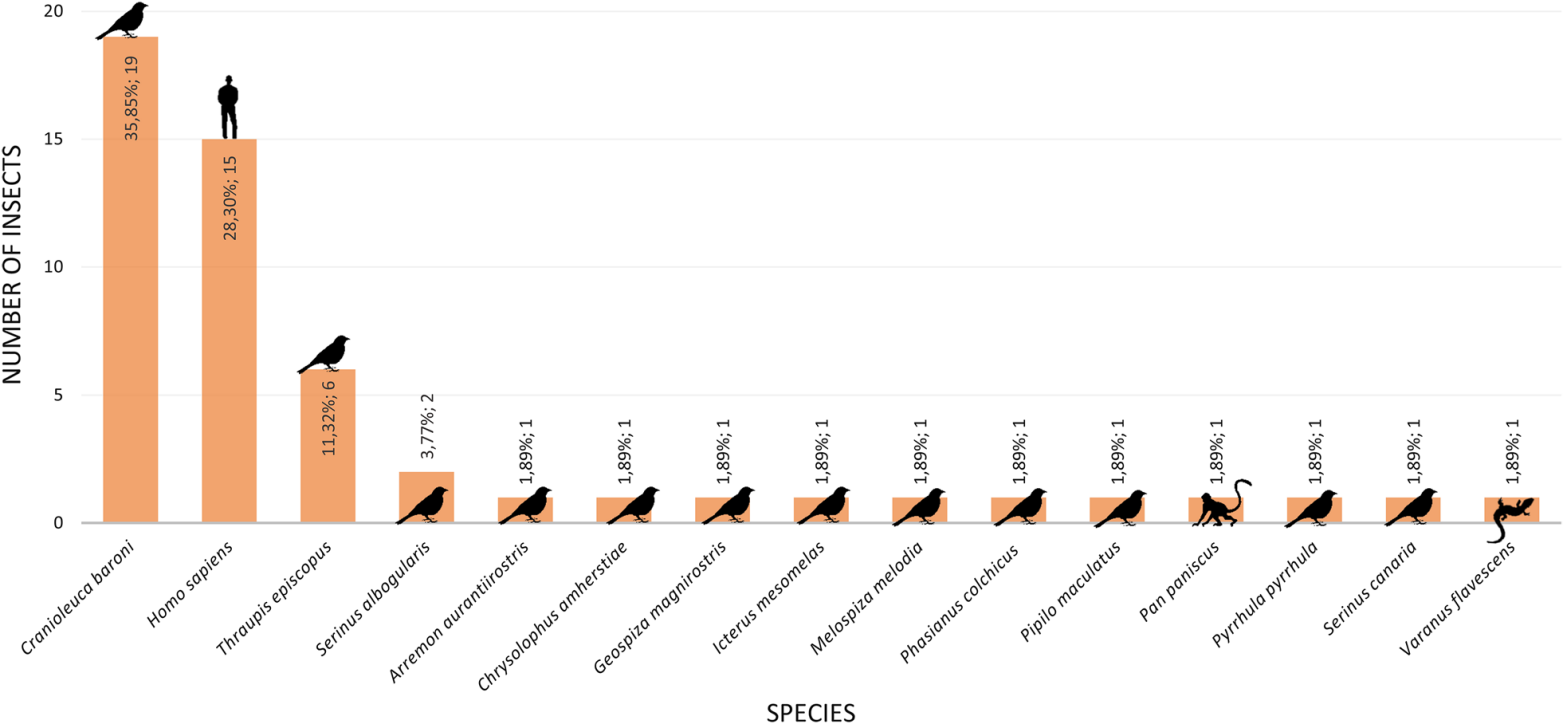
Frequency of the 15 feeding sources found in the evaluated *Ps. arthuri*

A total of 15 feeding sources of *Ps. arthuri* were found (Fig. 2). The frequencies were: *Cranioleuca baroni* (Baron’s spinetail) (35.85%, *n* = 19), *Homo sapiens* (human) (28.30%, *n* = 15), *Thraupis episcopus* (blue-gray tanager) (11.32%, *n* = 6), *Serinus albogularis* (white-throated canary) (3.77%, *n* = 2) and 11 other species corresponding to 1.89% [*Arremon aurantiirostris* (orange-billed sparrow), *Chrysolophus amherstiae* (Lady Amherst pheasant), *Geospiza magnirostris* (large ground-finch), *Icterus mesomelas* (yellow-tailed oriole), *Melospiza melodia* (song sparrow), *Phasianus colchicus alaschanicus* (common pheasant), *Pipilo maculatus* (spotted towhee), *Pan paniscus* (bonobo), *Pyrrhula pyrrhula* (common bullfinch), *Serinus canaria* (wild canary) and *Varanus flavescens* (yellow monitor)].

**Fig. 3:**
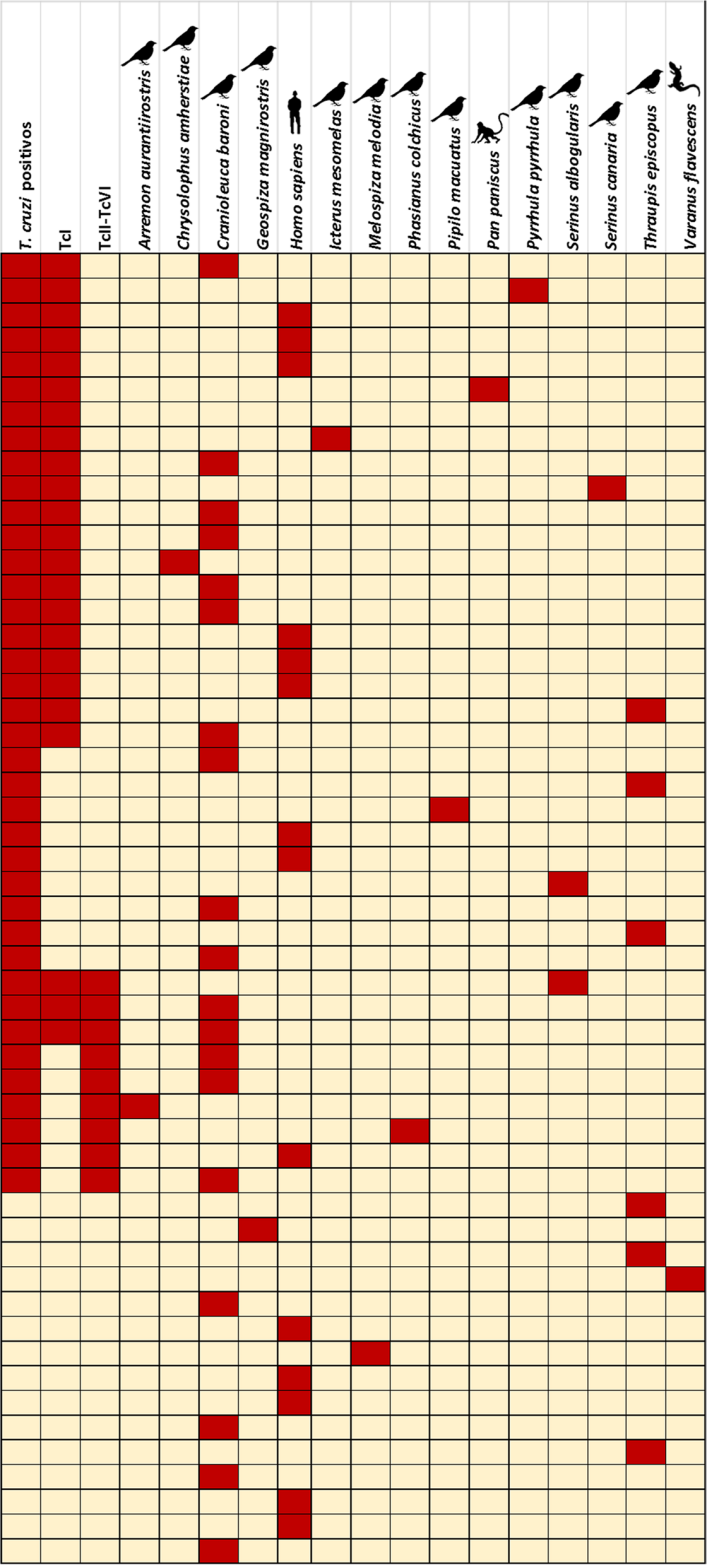
*T. cruzi* presence, DTUs and feeding sources of 53/60 *Ps. arthuri* collected

Additionally, we found that the majority of insects feeding on different bird species were *T. cruzi* positive, especially those who fed on *C. baroni*, and mostly presented TcI DTU. Additionally, we found that insects that were *T. cruzi* positive fed on humans presented TcI DTU (6/14) and just one insect had TcII. Mixed infection was found when *Ps. arthuri* fed on 2 species: *C. baroni* (2) and *S. albogularis* (1). Two species, *Arremon aurantiirostris* and *Phasianus colchicus alaschanicus*, presented only TcII. Some feeding sources were *T. cruzi* positive but no information about DTUs was available (Fig. 3).

### Proxy associations between *T. cruzi* infection and eco-epidemiological variables

**Fig. 4:**
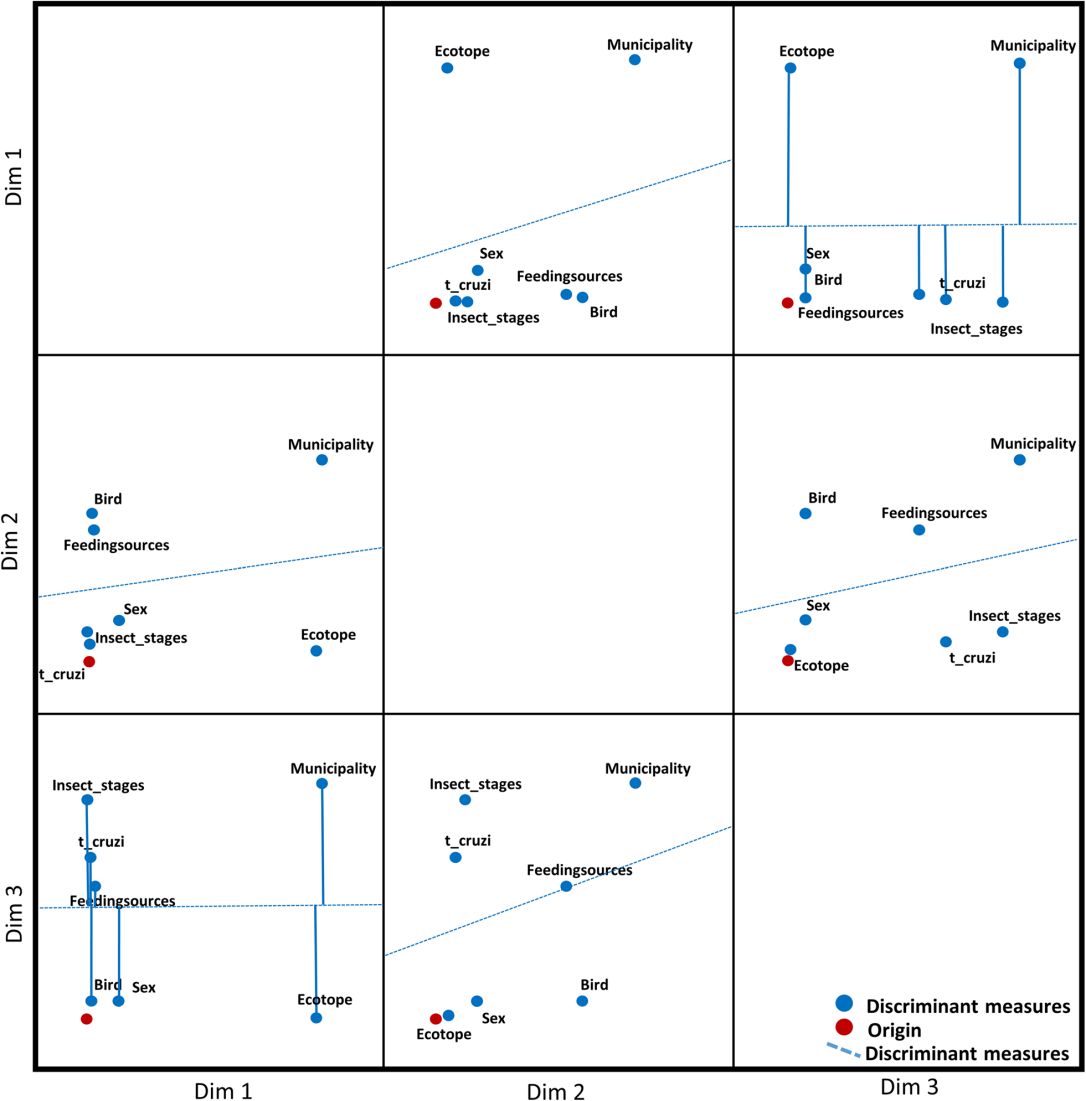
Proximity bi-plot between *T. cruzi* and the eco-epidemiological variables evaluated

Two subgroups of variables were identified through the MCA. The first was associated with the variables ecotope and locality, and the second was associated with the variables *T. cruzi* infection, insect stage, sex, bird nest and feeding source. Additionally, in dimension 2 we identified a geometric relation between the variable ecotope and *T. cruzi* presence (Table 2). Once MCA values were obtained, a proximity bidimensional plot (bi-plot) was made to elucidate relations between variables by representing the geometric distribution in three dimensions of evaluated variables (Fig. 4). It can be observed that, generally, *T. cruzi* presence was mostly associated with insect stage, sex, bird nest and feeding source. Logistic regression results showed that Pore municipality, adult insect stage, male *Ps. arthuri*, *C. cela* bird nest and *C. baroni* feeding preference were related the most with *T. cruzi* presence (Table 3). This analysis is consistent with results obtained in the MCA (Fig. 4).

**Table 2:** MCA values for each variable in 3 dimensions (Dim) as previously described

Variable	Dim 1	Dim 2	Dim 3
Locality (municipality)	1.349^a^	0.804	0.661
Ecotope	1.311^a^	0.041^c^	0.009
Insect stage	0.008^b^	0.116	0.613
Sex	0.190^b^	0.162	0.048
Bird nest	0.033^b^	0.590	0.050
Feeding sources	0.049^b^	0.524	0.371
*T. cruzi*	0.020^b^	0.074^c^	0.452

^a^First subgroup of variables

^b^Second subgroup of variables

^c^Relation between ecotope and *T. cruzi* presence

*Note*: α-Cronbach values: dim 1, 0.772; dim 2, 0.662; dim 3, 0.637. Inercy values: dim1, 0.423; dim 2, 0.330; dim 3, 0.315

**Table 3:** Significative variables obtained with logistic regression

Variable	Characteristic	β-coefficient	*P*-value	OR (Exp[B])	95% CI
Municipality	Pore, Casanare	2.398	0.022	11.0	1.42–85.2
Ecotope	Sylvatic	0.880	0.002	2.41	1.37–4.24
Insect stage*	Adult	1.030	0.001	2.80	1.55–5.04
Sex*	Male	1.145	0.008	3.14	1.34–7.35
Sex	Female	0.916	0.028	2.50	1.10–5.67
Bird nest*	*C. cela*	1.386	0.032	4.00	1.12–14.17
Bird nest	*P. rufifrons*	0.728	0.025	2.07	1.09–3.92
Feeding source*	*C. baroni*	1.322	0.019	3.75	1.24–11.29

*Note*: Response variable was presence (1) or ausence (2) of *T. cruzi*. Explicative variables are the ones shown in the table. A stratified simulation (1000 samples) was made for the response variable (*T. cruzi* presence) in each executed model. Wald estimator was used to determine the OR 95% confidence intervals

*Most statistically significant variables

## Discussion

In Colombia 15 of the 26 registered triatomines have been reported as naturally infected with *T. cruzi* and some are considered secondary vectors for *T. cruzi* transmission [18]. This last group includes *Ps. arthuri*, a triatomine with sylvatic habits [21]. *Psammolestes arthuri* has been reported in three departments of Colombia, Meta, Arauca and Casanare, with no available data about *T. cruzi* presence [18]. Its presence has been associated with *Phacellodomus rufifrons* nests [37]. Furthermore, there are some studies that report triatomine species with sylvatic habits in a domiciliation process due to deforestation and ecosystemic fragmentation. Therefore, these triatomines have begun to be considered an important factor in parasite transmission [18, 19], especially in Arauca and Casanare, where it has been found that sylvatic triatomine species such as *Ps. arthuri*, *P. geniculatus*, *T. maculata* and *T. venosa*, are moving to human houses [15, 28].

In this study, the percentage of *Ps. arthuri* infected with *T. cruzi* was high (70%), which might suppose a possibility that *Ps. arthuri* is changing its ornithophilic behavior to feed on other blood sources, including human blood (Fig. 2). It is important to highlight that niche changing or adaptation to other niches starts with a behavioral change [41], which is relevant in the context of new transmission scenarios of *T. cruzi*. By feeding solely on birds, triatomines cannot get infected with the parasite, because birds are naturally resistant to *T. cruzi* infection due to their innate immune mechanism against the parasite that quickly eliminates infective forms from their system [42]. Schaub [43] showed that chicken blood had a lytic effect for trypanosomes after an incubation period of 60 minutes, but transmission could still occur because of lysis-resistant epimastigotes in the triatomine gut. Otherwise, it is known that specific feeding preferences of triatomines can influence the transmission dynamics of *T. cruzi* [44]. A study showed that mice infected with *T. cruzi* isolates previously exposed to bird blood presented a high survival rate. This suggests that ornithophilic behavior of triatomines could modulate the parasite and bird blood does not prevent parasite development and transmission [45].

On the other hand, direct transmission of *T. cruzi* between insects can occur through entomophagy behavior such as cannibalism, coprophagy and cleptohaematophagy. Cannibalism in triatomines is rare and poorly demonstrated but coprophagic behavior is associated with the acquisition of microbe symbionts and is a possibility of infection that cannot be excluded [43]. Coprophagy has been described for many insects, such as Isoptera (termites), Hemiptera (true bugs) and Blattaria (cockroaches) as a predominant route of beneficial bacteria transmission [46], and the possibility of *T. cruzi* transmission should also be considered. Otherwise, some triatomine species are able to feed on the hemolymph of other insects if starving [47]. Sandoval et al. [48] showed that *Belminus herreri* in early stages was unable to feed on vertebrate hosts but successfully fed on replete *R. prolixus*, showing a cleptohaematophagy behavior. This behavior has also been reported with *R. prolixus* nymphs [49]. Therefore, this can be considered as another possible way by which *Ps. arthuri* could become infected with *T. cruzi*, taking into account that this triatomine has been found in sympatry with *R. prolixus* [25, 28], but further studies are required to prove this hypothesis.

Most of the *T. cruzi*-positive insects (70.7%, 29/43) were found in *P. rufifrons* nests (Table 2). The same as reported by Cruz-Guzmán et al. [25]. However, in Table 3, logistic regression indicates that the *C. cela* bird nest is statistically more significant with respect to the presence of *T. cruzi* (OR 4.00; 95% CI: 1.12–14.17) but this could be explained by the sample size in these nests. Additionally, other variables such as adult insect stage (OR 2.8; 95% CI: 1.55–5.04), male sex (OR 3.14; 95% CI: 1.34–7.35) and feeding on *C. baroni* (OR 3.75; 95% CI: 1.24–11.29) appear to be associated with the presence of *T. cruzi* in *Ps. arthuri*. Adult life stage association with *T. cruzi* presence in *Ps. arthuri* could be explained if it is considered as the mobile stage, in which triatomines can move around to take different meals. Regarding the *C. baroni* relation with *T. cruzi* presence, we consider it plausible that insects feeding on this bird species are more likely to feed on other animals that might be infected because these birds leave their nests (migrate), forcing triatomines to move and find other feeding sources [25].

In a study made in Venezuela in 2014, Cruz-Guzmán et al. [25] found a few *Ps. arthuri* naturally infected with *T. cruzi*, despite the fact that these triatomines fed on birds. This feeding preference is not a coincidence, because *Psammolestes* genus is closely related with *Rhodnius* (Rhodniini Tribe) [38], and many species of this genus feed on birds, due to their associations with palm trees in which there are bird nests [39]. Something similar occurs with *T. maculata*, a triatomine with wide distribution in Colombia, whose diet also consist mainly of bird blood. Because of that, this triatomine is excluded from vector control programs [40]. However, in 2016 Hernández et al. [15] reported *T. maculata* feeding on humans with a frequency of 75% and also infected with *T. cruzi* with a frequency of 67%. They also found TcI and TcII in some specimens, suggesting a connection between parasites’ domestic and sylvatic transmission cycles by this triatomine. Finally, they highlighted *T. maculata* as a potential vector for CD and underlined the importance of prioritizing secondary vectors in vector surveillance due to their capacity of domiciliation. These findings may suggest a future behavior that *Ps. arthuri* could develop, but further studies about its potential as a *T. cruzi* vector are needed. The MCA results highlight that *T. cruzi* presence appears to be highly associated with Pore municipality (Fig. 4). This is due to the sample size, because this was the only municipality in which all of the specimens, except one, were positive for *T. cruzi* presence. However, the obtained frequency for *T. cruzi* presence in Pore, Casanare, could be explained by taking into account other triatomines present in this area such as *R. prolixus*, a triatomine species with a wide distribution in Colombia and the one that presents the highest rate of infection with *T. cruzi* [18].

Triatomines are known as nest-dwelling insects, and their usual hosts are tree-dwelling animals such as birds, reptiles, marsupials and burrow vertebrates (e.g. bats, rodents and armadillos) [50]. Additionally, their alimentary habits may be influenced by the density and availability of new feeding sources [30] which is relevant in the context of human urbanization. Here, we highlight that 28.3% of *Ps. arthuri* that were positive for *T. cruzi* had fed on humans (Fig. 2), being the first study in Colombia and, to our knowledge, in the region to report it. This is a fact to underline, because there is an option where, besides the fact that *Ps. arthuri* is reaching dwellings, human activities near houses mean that people are accessible to these triatomines. Further studies are required to evaluate the possibility of *Ps. arthuri* effectively transmitting *T. cruzi* to humans. In this case, these findings become relevant for CD transmission; particularly in an endemic region for CD such as Casanare, which shows the highest incidence for this pathology in the country and where most of the specimens were collected.

The wide variety of feeding sources of *Ps. arthuri* found in this study could be explained because other animals often use *P. rufifrons* nests [51], which may explain the non-bird feeding sources found in some of the insects evaluated, such as *Varanus flavescens* and *Pan paniscus* (Fig. 2). Additionally, *Ps. arthuri* has been found in other bird nests and under the bark of dead trees [52], suggesting they are moving through different niches looking for blood meals. The description of feeding patterns of sylvatic triatomines is relevant for a good understanding of *T. cruzi* transmission and its circulation among different hosts. Furthermore, the presence of the parasite in a sylvatic vector considered as secondary for humans is highly relevant in the context of CD vector control programs, because in a hypothetic scenery where *R. prolixus* is eliminated, other triatomines with similar feeding behaviors could take their niche and then possibly transmit *T. cruzi* [30]. *In vivo* studies of *Ps. arthuri* are required to evaluate the progression and development of *T. cruzi* life-cycle. Additionally, it is important to evaluate defecation patterns, insect densities, parasite-triatomine interactions, triatomine microbiota, immune response and ecology, because these are main factors to determine if a triatomine could be a potential vector for *T. cruzi* [44, 53].

It is important to highlight the presence of the TcI_Dom_ genotype in 40.4% of the *Ps. arthuri* which were positive for *T. cruzi*, knowing that 97.6% of insects were collected in a sylvatic ecotope, indicating the intrusion of domestic DTUs in the sylvatic cycle of the parasite. In contrast, Cruz-Guzmán et al. [25] reported the presence of TcIII in an adult specimen of *Ps. arthuri*. This DTU belongs to the sylvatic cycle of the parasite and has been found in armadillos and didelphimorphos [54], animals that are frequently found near dwellings. Another study of the secondary vectors of CD, reported *T. maculata* and *P. geniculatus* infected with TcI/TcIII and TcI-TcV, respectively, concluding that this triatomine represents an important connection between sylvatic and domestic transmission cycles, facilitating the circulation of many DTUs [15]. In this study we did not discriminate DTUs from the TcII-TcVI group. Further studies are required to determine truly circulating genotypes. Futhermore, TcI is associated with arboricoral mammals such as *Didelphis marsupialis* and others like *Rattus rattus* and *Canis lupus familiaris* [5, 15, 16], while TcIII and TcIV are related to armadillos [55]. *Psammolestes arthuri* may be feeding on these reservoirs associated with the domestic cycle, explaining the presence of TcI and TcII-TcVI DTUs. Furthermore, the presence of domestic DTUs may be explained considering that collection sites are located near human settlements (Fig. 1b) and that *Ps. arthuri* could be moving through domestic and sylvatic transmission cycles, as reported in vectors and synanthropic reservoirs for the parasite [10]. These triatomines could be circulating between sylvatic and domestic ecotope not only because of the need to find new feeding sources, but through active dispersal, because they might be attracted to the artificial light of human dwellings [56]. This phenomenon has important epidemiological significance, because if they are truly attracted to this light, the probability of triatomines attracted to a dwelling increases [47]. Jácome-Pinilla et al. [57] studied the associated risks among dispersive nocturnal flights of sylvatic triatomines because of artificial lights in northeastern Colombia. They reported *Ps. arthuri*, *T. maculata*, *P. geniculatus* and *R. prolixus* being attracted to light traps, highlighting a potential risk of active dispersion of sylvatic triatomines and their implications in the introduction of sylvatic DTUs into the domestic transmission cycle. Moreover, a study in Brazil [58] about the attraction of Chagas disease vectors to artificial light found that almost all of the known vectors of CD in the zone were attracted by artificial light sources, and they propose this as a possible route by which triatomines can reach dwellings and become involved in *T. cruzi* transmission.

## Conclusions

We present herein the first *Ps. arthuri* study in Colombia regarding *T. cruzi* infection and its feeding preferences. Our findings indicate that *Ps. arthuri* is feeding on other potential reservoirs for *T. cruzi* aside from birds and could possibly be maintaining the zoonotic cycle of the parasite. Furthermore, the finding that these triatomines are feeding on humans may be highly relevant for the epidemiology and control of CD, but further studies are needed to evaluate *Ps. arthuri* as a potential vector for *T. cruzi*. These should consider factors such as the biology of the parasite inside the triatomine, changes that the parasite can trigger in the host and feeding and defecation behavior, because these are important factors to better understand *T. cruzi* transmission. Moreover, studies using other molecular techniques, such as deep sequencing, are required to improve feeding source detection and *T. cruzi* genotyping. Additionally, a wider sampling is needed to determine true associations of *Ps. arthuri* and the presence of *T. cruzi*. Finally, we encourage the scientific community to keep including other triatomine species into CD eco-epidemiological studies. This might help to develop a better understanding of transmission dynamics of the parasite. Also, we encourage the government to pay special attention to *Ps. arthuri* in eastern Colombia, considering the ability of triatomines to adapt to new environments and the findings of the present study.

## Additional file

**Additional file 1: Figure S1.** Model selection using SPSS software modeler.

## Abbreviations

CD: Chagas disease
DTU: discrete typing unit
PCR: polymerase chain reaction
qPCR: real-time PCR
SL-IR: spliced leader-intergenic region
MCA: multiple correspondence analysis
OR: odds ratio

## References

[1] Rassi A, Rassi A, Marcondes de Rezende J (2012). American trypanosomiasis (Chagas disease). Infect Dis Clin North Am..

[2] WHO. Chagas disease (American trypanosomiasis). 2018. https://www.who.int/en/news-room/fact-sheets/detail/chagas-disease-(american-trypanosomiasis). Accessed 20 Nov 2018.

[3] WHO. Weekly epidemiological record Relevé épidémiologique hebdomadaire. 2015. http://www.who.int/wer/2015/wer9006.pdf?ua=1. Accessed 20 Nov 2018.

[4] OPS. Guía de Atención Clínica de la enfermedad de Chagas. Ministerio de la Protección Social, Republica de Colombia, Organización Panamericana de la Salud; 2010. Accessed 20 Nov 2018.

[5] Guhl F, Ramírez JD (2011). *Trypanosoma cruzi* I diversity: towards the need of genetic subdivision?. Acta Trop..

[6] Zingales B, Miles MA, Campbell DA, Tibayrenc M, Macedo AM, Teixeira M (2012). The revised *Trypanosoma cruzi* subespecific nomenclature: rationale, epidemiological relevance and research applications. Infect Genet Evol..

[7] Marcili A, Lima L, Cavazzana M, Junqueira AC, Veludo HH, Da Silva M (2009). A new genotype of *Trypanosoma cruzi* associated with bats evidenced by phylogenetic analyses using *SSU* rDNA, cytochrome b and Histone H2B genes and genotyping based on ITS1 rDNA. Parasitology..

[8] Pinto CM, Kalko EKV, Cottontail I, Wellinghausen N, Cottontail VM (2012). TcBat a bat - exclusive lineage of *Trypanosoma cruzi* in the Panama Canal Zone, with comments on its classification and the use of the *18S* rRNA gene for lineage identification. Infect Genet Evol..

[9] Ramírez JD, Hernández C, Montilla M, Zambrano P, Flórez AC, Parra E, Cucunubá ZM (2014). First report of human *Trypanosoma cruzi* infection attributed to TcBat genotype. Zoonoses Public Health..

[10] Ramírez JD, Hernández C (2018). *Trypanosoma cruzi* I: towards the need of genetic subdivision? Part II. Act Trop..

[11] Parra-Henao G, Suárez-Escudero LC, González-Caro S (2016). Potential distribution of Chagas disease vectors (Hemiptera: Reduviidae, Triatominae) in Colombia, based on ecological niche modeling. J Trop Med.

[12] Gordon ERL, Georgieva AY, Weirauch C (2017). Sylvatic host associations of Triatominae and implications for Chagas disease reservoirs: a review and new host records based on archival specimens. PeerJ.

[13] Herrera L (2010). Una revisión sobre reservorios de *Trypanosoma* (*Schizotrypanum*) *cruzi* (Chagas, 1909), agente etiológico de la enfermedad de Chagas. Bol Malariol y Salud Ambient..

[14] Rendón LM, Guhl F, Cordovez JM, Erazo D (2015). New scenarios of *Trypanosoma cruzi* transmission in the Orinoco region of Colombia. Mem Inst Oswaldo Cruz..

[15] Hernández C, Salazar C, Brochero H, Teherán A, Buitrago LS, Vera M (2016). Untangling the transmission dynamics of primary and secondary vectors of *Trypanosoma cruzi* in Colombia: parasite infection, feeding sources and discrete typing units. Parasit Vectors..

[16] León CM, Hernández C, Montilla M, Ramírez JD (2015). Retrospective distribution of *Trypanosoma cruzi* I genotypes in Colombia. Mem Inst Oswaldo Cruz..

[17] Cantillo-Barraza O, Gómez-Palacio A, Salazar D, Mejía-Jaramillo AM, Calle J, Triana O (2010). Distribución geográfica y ecoepidemiología de la fauna de triatominos (Reduviidae: Triatominae) en la Isla Margarita del departamento de Bolívar, Colombia. Biomédica.

[18] Guhl F, Aguilera G, Pinto N, Vergara D (2007). Actualización de la distribución geográfica y ecoepidemiológica de la fauna de triatominos (Reduviidae: Triatominae) en Colombia. Biomédica..

[19] Wolff M, Castillo D (2000). Evidencias de domesticación y aspectos biológicos de *Panstrongylus geniculatus* (Latreille, 1811) (Hemiptera: Reduviidae). Acta Ent Chilena..

[20] Ramírez-Mejía D, Mendoza E (2010). El papel funcional de la interacción planta-mamífero en el mantenimiento de la diversidad tropical. Biológicas..

[21] Abad Franch F, Monteiro F, Jaramillo N, Gurgel-Gonҫalves R, Braga Stehling Dias F, Diotaiuti L (2009). Ecology, evolution, and the long-term surveillance of vector-borne Chagas disease: a multi-scale appraisal of the tribe Rhodniini (Triatominae). Act Trop..

[22] Pifano C (1938). Anotaciones acerca de *Psammolestes arthuri* Pinto, 1926 (Hemiptera, Heteroptera: Triatominae), reduvideo hematófago encontrado en nidos de cucarachero de monte (probablemente Dendrocolaptidae) en un sector del valle del Yaracuy. Su importancia como posible vector en la naturaleza del *Schizotripanum cruzi* Chagas, 1909.. Gac Méd Caracas..

[23] Pifano F (1909). Nota sobre la infestación experimental y en la naturaleza del *Psammolestes arthuri* (Pinto, 1926) por el *Schizotrypanum cruzi* Chagas, 1909. 440 *Psammolestes arthuri* naturalmente infectado con *Trypanosoma cruzi*. Gac Med Caracas..

[24] Sherlock I, Carcavallo R, Galíndez I, Carcavallo R, Galíndez I, Jurberg J, Lent H (1997). List of natural and experimental flagellate infections in several triatominae species. Atlas of Chagas disease vectors in the Americas.

[25] Cruz-Guzmán PJ, Morocoima A, Chique JD, Ramonis-Quintero J, Toquero-Uzacátegui M, Carrasco HJ (2014). *Psammolestes arthuri* naturally infected with *Trypanosoma cruzi* found in sympatry with *Rhodnius prolixus* and *Triatoma maculata* on bird nests in Anzoátegui, Venezuela. Saber Univ Oriente..

[26] Molina J, Gualdrón L, Brochero H, Olano V, Barrios D, Guhl F (2000). Distribución actual e importancia epidemiológica de las especies de triatominos (Reduviidae: Triatominae) en Colombia. Biomédica.

[27] Kierszembaum F, Ivanyi J, Budzco D (1976). Mechanisms of natural resistance to trypanosomal infection. Role of complement in avian resistance to *T cruzi* infection. Immunology..

[28] Angulo VM, Esteban L, Luna KP (2012). *Attalea butyracea* próximas a las viviendas como posible fuente de infestación domiciliaria por *Rhodnius prolixus* (Hemiptera: Reduviidae) en los Llanos Orientales de Colombia. Biomédica..

[29] Cazorla-Perfetti D (2015). *Psammolestes arthuri* naturally infected with *Trypanosoma cruzi* found in sympatry with *Rhodnius prolixus* and *Triatoma maculata* on bird nests in Anzoátegui, Venezuela. Saber Univ Oriente Venezuela..

[30] Rabinovich L, Kitron U, Obed Y, Yoshioka M, Gottdenker N, Chaves L (2011). Ecological patterns of blood-feeding by kissing-bugs (Hemiptera: Reduviidae: Triatominae). Mem Inst Oswaldo Cruz..

[31] Duffy T, Cura CI, Ramírez JC, Abate T, Cayo NM, Parrado R (2013). Analytical performance of a multiplex real-time PCR assay using TaqMan probes for quantification of *Trypanosoma cruzi* satellite DNA in blood samples. PLoS Negl Trop Dis.

[32] Ramírez JD, Guhl F, Umezawa ES, Morillo C, Rosas F, Marin-Neto J (2009). Evaluation of adult chronic Chagas’ heart disease diagnosis by molecular and serological methods. J Clin Microbiol..

[33] Souto RP, Fernandes O, Macedo AM, Campbell D, Zingales B (1996). DNA markers define two major phylogenetic lineages of *Trypanosoma cruzi*. Mol Biochem Parasitol..

[34] Herrera C, Bargues MD, Fajardo A, Montilla M, Triana O, Vallejo GA, Guhl F (2007). Identifying four *Trypanosoma cruzi* I isolate haplotypes from different geographic regions of Colombia. Infect Genet Evol..

[35] Dumonteil E, Ramírez-Sierra MJ, Pérez-Carillo S, Teh-Poot C, Herrera C, Gourbiére S, Waleckx E (2018). Detailed ecological associations of triatomines revealed by metabarcoding and next-generation sequencing: implications for triatomine behavior and *Trypanosoma cruzi* transmission cycles. Sci Rep..

[36] Kumar S, Stecher G, Li M, Knyaz C, Tamura K (2018). MEGA X: Molecular Evolutionary Genetics Analysis across computing platforms. Mol Biol Evol..

[37] Barreto M, Barreto P, D’Alessandro A (1984). *Psammolestes arthuri* (Hemiptera: Reduviidae) and its parasite *Telenomus capito* (Hymenoptera: Scelionidae) in Colombia. J Med Entomol.

[38] Schofield Christopher J (2000). Biosystematics and evolution of the Triatominae. Cad Saúde Públ..

[39] Gaunt M, Miles MA (2000). The ecotopes and evolution of triatomine bugs (Triatominae) and their associated trypanosomes. Mem Inst Oswaldo Cruz.

[40] Luitgards-Moura JF, Vargas AB, Almeida CE, Magno-Esperança G, Agapito-Souza R, Folly-Ramos E (2005). A *Triatoma maculata* (Hemiptera: Reduviidae, Triatominae) population from Roraima, Amazon Region, Brazil, has some bionomic characteristics of a potential Chagas disease vector. Rev Inst Med Trop Sao Paulo..

[41] Mayr E (1963). Animal species and evolution.

[42] Kierszembaum F, Gottlieb CA, Budzko DB (1981). Antibody-independent, natural resistance of birds to *Trypanosoma cruzi* infection. J Parasitol.

[43] Schaub GA (1988). Direct transmission of “*Trypanosoma cruzi*” between vectors of Chagas’ disease. Acta Trop..

[44] De Fuentes-Vicente J, Gutiérrez-Cabrera A, Flores-Villegas A, Lowenberg C, Benelli G, Salazar-Schettino P, Córdoba-Aguilar A (2018). What makes an effective Chagas disease vector? Factors underlying *Trypanosoma cruzi*-triatomine interactions. Acta Trop..

[45] Calderon-Arguedas O (2003). Variaciones biológicas de *Trypanosoma cruzi* (Kinetoplastida: Trypanosomatiade) asociadas con la ingestión de diferentes tipos de sangre por el vector *Triatoma dimidiata* (Hemiptera: Reduviidae). Parasitol Latinoam..

[46] Salem H, Florez L, Gerardo N, Kaltenpoth M (2015). An out-of-body experience: the extracellular dimension for the transmission of mutualistic bacteria in insects. Proc R Soc B..

[47] Lazzari CR, Pereira MH, Lorenzo MG (2013). Behavioural biology of Chagas disease vectors. Mem Inst Oswaldo Cruz.

[48] Sandoval CM, Joya MI, Gutierez R, Angulo VM (2000). Cleptohaematophagy of the triatomine bug *Belminus herreri*. Med Vet Entomol.

[49] Piñero DF, Carcavallo RU, Fernandez E (1988). Canibalismo y transmisión directa de *Trypanosoma cruzi* entre ninfas de *Rhodnius prolixus*. Chagas.

[50] Otálora-Luna F, Pérez-Sánchez AJ, Sandoval C, Aldana E (2015). Evolution of hematophagous habit in Triatominae (Heteroptera: Reduviidae). Rev Chil Hist Nat.

[51] Skutch AF (1969). A study of the rufous-fronted thornbird and associated birds, Part II: birds which breed in thornbirdsʼ nests. Wilson Bull.

[52] Lent H, Wygodzinsky P (1979). Revision of the triatomine (Hemiptera: Reduviidae) and their significance as vectors of Chagas’ disease. Bull Am Mus Nat Hist..

[53] Zeledón R, Alvarado R, Jirón LF (1977). Observations on the feeding and defecation patterns of three triatomine species (Hemiptera: Reduviidae). Acta Trop..

[54] Morocoima A, Carrasco HJ, Boadas J, Chique JD, Herrera L, Urdaneta-Morales S (2012). *Trypanosoma cruzi* III from armadillos (*Dasypus novemcinctus novemcinctus*) from northeastern Venezuela and its biological behavior in murine model. Risk of emergency of Chagas’ disease. Exp Parasitol.

[55] Yeo M, Acosta N, Llewellyn M, Sánchez H, Adamson S, Miles GA (2005). Origins of Chagas disease: *Didelphis* species are natural hosts of *Trypanosoma cruzi* I and armadillos hosts of *Trypanosoma cruzi* II, including hybrids. Int J Parasitol.

[56] Vazquez-Prokopec GM, Ceballos LA, Kitron U, Gurtler RE (2004). Active dispersal of natural populations of *Triatoma infestans* (Hemiptera: Reduviidae) in rural northwestern Argentina. J Med Entomol..

[57] Jácome-Pinilla D, Hincapie-Peñaloza E, Ortiz MI, Ramírez JD, Guhl F, Molina J (2015). Risks associated with dispersive nocturnal flights of sylvatic Triatominae to artificial lights in a model house in the northeastern plains of Colombia. Parasit Vectors..

[58] Castro, Marcelo CM, Barrett, Toby V, Santos, Walter S, Abad-Franch F, Rafael JA (2010). Attraction of Chagas disease vectors (Triatominae) to artificial light sources in the canopy of primary Amazon rainforest. Mem Inst Oswaldo Cruz..

