# Supplementary figures and images for: Trypanosoma cruzi infection, discrete typing units and feeding sources among Psammolestes arthuri (Reduviidae: Triatominae) collected in eastern Colombia
Source: Parasit Vectors. 2019 Apr 8;12:157. doi: 10.1186/s13071-019-3422-y (PMC6454608; doi:10.1186/s13071-019-3422-y)

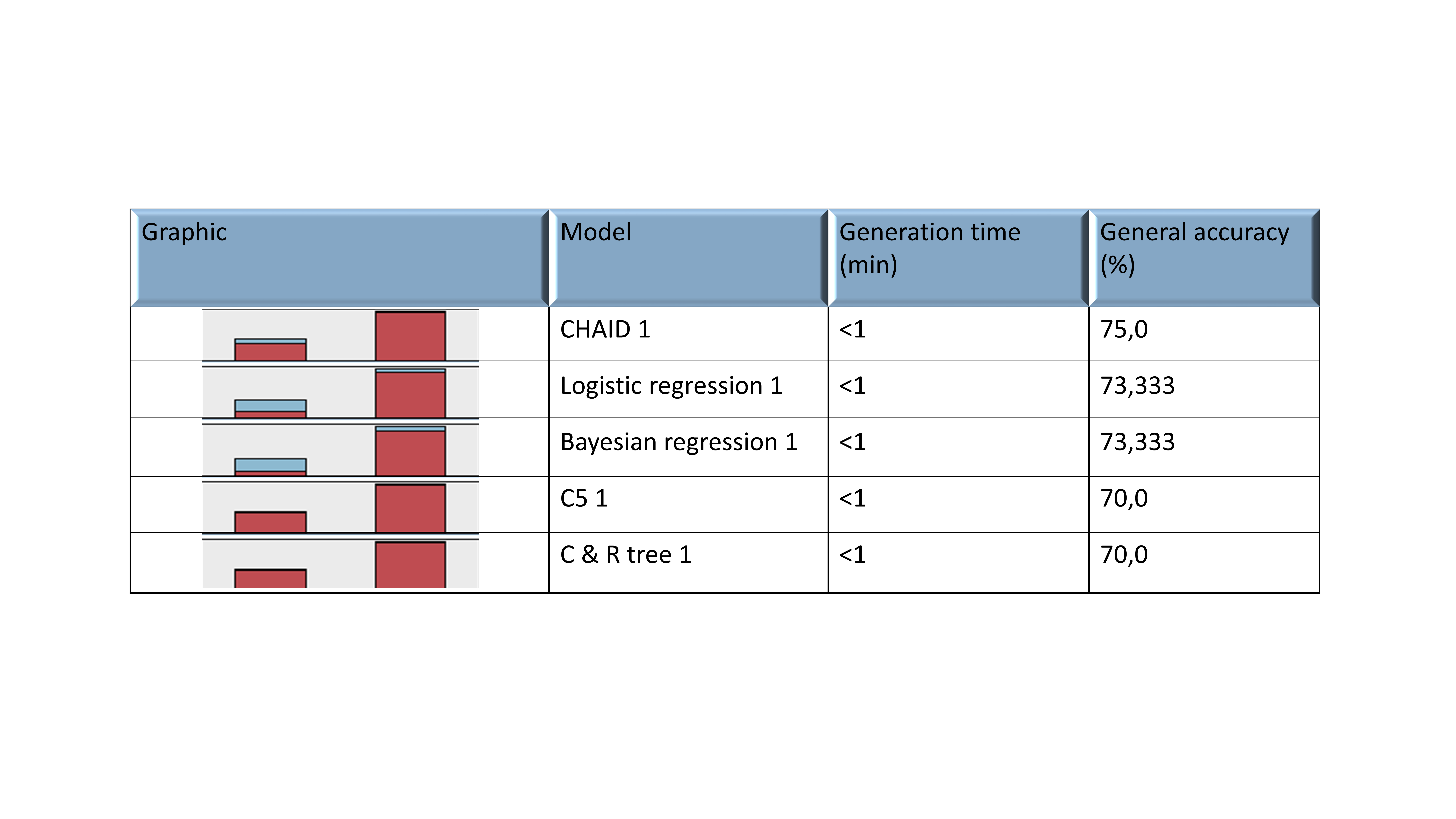

Supplement: Supplementary file 1 — Additional file 1: Figure S1. Model selection using SPSS software modeler. [file 13071_2019_3422_MOESM1_ESM.tif]
